# Altered myogenesis and premature senescence underlie human TRIM32-related myopathy

**DOI:** 10.1186/s40478-019-0683-9

**Published:** 2019-03-01

**Authors:** E. Servián-Morilla, M. Cabrera-Serrano, E. Rivas-Infante, A. Carvajal, P. J. Lamont, A. L. Pelayo-Negro, G. Ravenscroft, R. Junckerstorff, J. M. Dyke, S. Fletcher, A. M. Adams, F. Mavillard, M. A. Fernández-García, J. L. Nieto-González, N. G. Laing, C. Paradas

**Affiliations:** 1 Neuromuscular Disorders Unit, Department of Neurology, Instituto de Biomedicina de Sevilla, Hospital U. Virgen del Rocío/CSIC/Universidad de Sevilla, Sevilla, Spain; 2 Centro de Investigación Biomédica en Red sobre Enfermedades Neurodegenerativas (CIBERNED), Madrid, Spain; 3 Centre for Medical Research, University of Western Australia, Harry Perkins Institute of Medical Research, Perth, Australia; 4 Department of Neuropathology, Hospital U. Virgen del Rocío/ Instituto de Biomedicina de Sevilla (IBiS), Sevilla, Spain; 5 Neuromuscular Unit, Hospital Virgen de las Nieves, Granada, Spain; 6 Neurology Department, University Hospital Marqués de Valdecilla (IDIVAL), Santander, Cantabria Spain; 7 PathWest Laboratory Medicine WA, Section of Neuropathology, Royal Perth Hospital, Perth, WA Australia; 8 Centre for Comparative Genomics, Murdoch University, Perth, 6150 Australia; 9 Perron Institute for Neurological and Translational Science, University of Western Australia, Nedlands, Australia; 10 Neurology Department, Health in Code S.L., A Coruña, Spain; 11 Department of Medical Physiology and Biophysics, Instituto de Biomedicina de Sevilla, Hospital U. Virgen del Rocío/CSIC/Universidad de Sevilla, Sevilla, Spain

**Keywords:** Muscle dystrophy, TRIM32, E3 ubiquitin -ligase, Proliferation/differentiation, Autophagy

## Abstract

TRIM32 is a E3 ubiquitin -ligase containing RING, B-box, coiled-coil and six C-terminal NHL domains. Mutations involving NHL and coiled-coil domains result in a pure myopathy (LGMD2H/STM) while the only described mutation in the B-box domain is associated with a multisystemic disorder without myopathy (Bardet-Biedl syndrome type11), suggesting that these domains are involved in distinct processes. Knock-out (T32KO) and knock-in mice carrying the c.1465G > A (p.D489N) involving the NHL domain (T32KI) show alterations in muscle regrowth after atrophy and satellite cells senescence. Here, we present phenotypical description and functional characterization of mutations in the RING, coiled-coil and NHL domains of TRIM32 causing a muscle dystrophy. Reduced levels of TRIM32 protein was observed in all patient muscle studied, regardless of the type of mutation (missense, single amino acid deletion, and frameshift) or the mutated domain. The affected patients presented with variable phenotypes but predominantly proximal weakness. Two patients had symptoms of both muscular dystrophy and Bardet-Biedl syndrome. The muscle magnetic resonance imaging (MRI) pattern is highly variable among patients and families. Primary myoblast culture from these patients demonstrated common findings consistent with reduced proliferation and differentiation, diminished satellite cell pool, accelerated senescence of muscle, and signs of autophagy activation.

## Introduction

Tripartite motif-containing protein 32 (TRIM32) is an E3 ubiquitin ligase, whose activity is contained in the RING finger domain of the tripartite TRIM/RBCC motif (RING, B-box, coiled-coil) [20, 30]. In addition, TRIM32 has six C-terminal NHL domains. The majority of the mutations described in *TRIM32* are clustered in the C-terminal NHL domain, which is defined by amino acid sequence homologies to regions of Ncl-1, HT2A and Lin-41 proteins, supporting its role in protein-protein interactions, critical for the ubiquitination process [13, 29, 44]. Mutations involving the NHL and coiled-coil domains are associated with limb-girdle muscular dystrophy 2H (LGMD2H) and sarcotubular myopathy (STM), which are considered as a continuum [15, 21]. Until now, only one mutation has been described involving the B-box domain, resulting in a different multisystemic disorder called Bardet-Biedl syndrome (BBS) type 11 with no skeletal muscle involvement, in an only family with four affecting siblings [7]. No mutations in the RING finger domain have been reported.

Proximal weakness is the characteristic feature of LGMD2H/STM, although other clinical findings, such as facial, axial or distal weakness, can be associated [5, 15, 19, 28, 33, 34, 39, 42]. Pathologically LGMD2H/STM are characterized by segmental vacuolation of the sarcoplasmic reticulum and transverse tubules [42], however vacuoles containing basophilic material consistent with autophagic vacuoles have also been observed in the muscle biopsy of these patients [21, 28]. The mutation c.1459G > A/p.D487N in the *TRIM32* gene, identified as a founder mutation in Hutterite population, has been the most frequently reported [15], but a recent series of 12 non-Hutterite patients with a TRIM32-related myopathy, with mutations located both in the NHL and coiled-coil domains, has been described [21].

A yeast model has shown that *TRIM32* mutations involving the NHL domain introduce conformational changes that impair the interaction properties of the protein, and consequently the ubiquitination process [39]. The most relevant mechanistic studies have been performed in the *Trim32* knockout (T32KO) and the knock-in mice carrying the Hutterite mutation (T32KI) [25, 26]. TRIM32, as a ubiquitous E3 ubiquitin ligase, has been demonstrated to promote degradation of several targets [1, 8, 18, 22, 24, 29, 31, 37], so the absence or abnormal function of TRIM32 due to recessive mutations would lead to loss of ubiquitination and accumulation of the TRIM32 substrates. E3 small ubiquitin-related modifier (SUMO) ligase (PIAS4) [1] and N-myc down-regulated protein 2 (NDRG2) have been previously identified as important TRIM32 substrates. Overexpression of PIAS4 is implicated in regulation of cellular senescence [4] and TRIM32-deficient primary myoblasts from T32KO mice have been demonstrated to undergo premature senescence and impaired myogenesis due to accumulation of PIAS4 [23]. On the other hand, NDRG2 overexpression in C2C12 myoblasts reduces cell proliferation and delayed cell cycle withdrawal during differentiation [14, 31]. Altogether, these results coming from cell and animal models, support the hypothesis that TRIM32 is involved in control of myogenesis and that premature senescence underlies myopathy in LGMD2H.

T32KI mice demonstrated that the Hutterite mutation (p.D489N) causes TRIM32 protein degradation, but this finding has not been reported in muscle from patients carrying missense mutations [25]. There is evidence that the EI24 autophagy-associated transmembrane protein is involved in the degradation of RING E3 ligases using the autophagy machinery, establishing a connection between two major protein degradation mechanisms, autophagy and the ubiquitin-proteasome system [11]. However, no specific studies about the role of autophagy in the degradation of TRIM32 have been reported.

We aimed to establish if patients with muscular dystrophy due to *TRIM32* mutations show evidence for the pathogenic mechanism postulated in the mouse models. This would provide a potential set of functional assays. We studied the muscle and myoblasts from three families with mutations in the NHL, coiled-coil and RING domains of TRIM32 resulting in a null-phenotype. We demonstrated common findings consistent with premature senescence, reduced myoblast proliferation and differentiation and, in addition, signs of autophagy activation seemingly involved in the degradation of the mutated TRIM32.

## Materials and methods

### Genetic work-up

DNA was extracted from blood using standard procedures. Patients were studied using next generation sequencing (NGS): patient IV.3/Family A and patient II.2/Family C with a panel of 34 genes associated with autosomal recessive limb-girdle muscular dystrophies, and patient II.3/Family B with a panel of 256 neuromuscular disease genes using HighSeq and NextSeq Illumina platforms. Affected and unaffected relatives of the probands were screened for their family mutations by Sanger sequencing.

### Histological studies

Muscle samples were obtained by open muscle biopsies from the biceps brachii muscle of the three patients from Family A (III.2, IV.3 and III.3), two patients from Family B (II.2 and II.3), one patient from Family C (II.2), five aged-matched healthy controls and four age-matched disease controls (LGMD1B, LGMD1C, LGMD2N, NEM6). Frozen muscle sections were stained for standard histological and histochemical techniques including hematoxylin and eosin (H&E) and neonatal myosin heavy chain (MHC-neo). Stained sections were evaluated with an Olympus BX41 (Tokyo, Japan) equipped with a ColorView IIIu camera (Olympus).

### Primary cell culture

Myoblasts from Family A (III.2 and IV.3), Family B (II.2 and II.3) and seven aged-matched healthy controls and three age-matched disease controls (LGMD2B, and X-EDMD) were used for the experiments. Fresh muscle samples were minced and cultured in a monolayer. Briefly, the culture medium for the myoblast stage (proliferation medium) contains 75% Dulbecco’s minimal essential medium (DMEM, Invitrogen) and 25% M199 medium (Invitrogen), supplemented with 10% fetal bovine serum (FBS), 10 μg/ml insulin, 2 mM glutamine, 100 units/ml penicillin, 100 μg/ml streptomycin, 0.25 μg/ml fungizone, 10 ng/ml epidermal growth factor, and 25 ng/ml fibroblast growth factor. To obtain highly purified myoblasts, each 10^7^ cells were mixed with 20 μl of CD56-coated microbeads (Milteny Biotec, Bergisch Gladbach, Germany) and incubated at 4 °C for 15 min. Unbound microbeads were removed by washing cells in excess PBS buffer followed by centrifugation at 400×g for 10 min. The cell pellet was resuspended in PBS buffer to a concentration of 2 × 10^8^ cells/ml before separation on a midiMACS cell separator (Milteny Biotec, Bergisch Gladbach, Germany).

### Transmission Electron microscopy (TEM)

A piece of muscle from each biopsy and primary myoblast from patients and controls were fixed in 2,5% glutaraldehyde in 0,1 M sodium cacodylate buffer (pH 7,4) for 3 h at 4 °C, washed with the same buffer, postfixed in 1% osmium tetraoxide in 0,1 M cacodylate buffer (pH 7,4) for 1 h at 4 °C, and then rinsed with distilled water. Samples were immersed in 2% uranyl acetate, dehydrated through a gradient acetone series (50, 70, 90 and 100%), and embedded in Spurr’s resin. Semithin sections (450 nm thickness) were obtained with a glass knife and stained with 1% toluidine blue for muscle fiber/myoblast localization and reorientation using a conventional optic microscope. Once a suitable block face of the selected area was trimmed, ultrathin sections (70 nm) were obtained using an ultramicrotome (Leica UC7) equipped with a diamond knife (Diatome) and collected on 200 mesh copper grids. Sections were viewed with a Zeiss Libra 120 transmission electron microscope (Carl Zeiss NTS GmbH, Oberkochen, Germany).

### Scanning Electron microscopy (SEM)

Primary myoblasts from patients and controls were fixed with 2.5% glutaraldehyde in 0.1 M sodium cacodylate buffer (pH 7.4) for 3 h at 4 °C and postfixed in 1% osmium tetraoxide in 0,1 M cacodylate buffer (pH 7.4) for 1 h at 4 °C. Samples were then dehydrated through a graded series of acetone solutions, critical point dried, gold sputtered, and examined with a Zeiss Auriga Field Emission Scanning Electron Microscope (Carl Zeiss NTS GmbH, Oberkochen, Germany).

### Immunohistochemical studies

Cultured primary myoblasts from patients and controls were fixed with 4% paraformaldehyde for 30 min, permeabilized in 0.2% Triton X-100 for 10 min and incubated in 1% BSA/PBS for 45 min. The following primary antibodies were used: rabbit polyclonal anti-TRIM32 (NBP1–33737) (1:500; Novus Biologicals); rabbit monoclonal anti-Desmin (D93FD5) (1:100; Abcam); mouse monoclonal anti-Ki67 (B56) (1:200; BD Biosciences); mouse monoclonal anti-Myogenin (FD5) (1:100; Abcam) and incubated for 12 h at 4 °C. Frozen muscle samples were fixed with 4% PFA for 15 min and immersed in citrate buffer (pH 6) for 30 min at 80 °C and washed with PBS. Afterward, muscle samples were blocked with 2% non-fat milk + 0.3% Triton X-100 in PBS for 30 min and with 5% BSA + 0.5% Triton X-100 in PBS for 1 h. The following primary antibodies were used: mouse monoclonal anti-Pax7 (1:50; DHSB); mouse monoclonal anti-collagen VI (3C4) (1:1000; Chemicon) and incubated for 3 days at 4 °C. After washing, samples were incubated with the secondary antibodies conjugated with Cy2 and Cy3 (1:500; Jackson ImmunoResearch) for 1 h. Finally, the nuclei were stained for 20 min with To-pro-3-Iodide (Topro 3) at 1/1000 in PBS and the slides were coverslipped with fluorescence mounting medium (Dako). Images were acquired on a Zeiss LSM 710 confocal laser scanning microscope using 20 x objective with a numerical aperture of 1.3. Images from controls and patients were obtained at the same day and under equal conditions (laser intensities and photomultiplier voltages). Maximal projections of Z-stacked images were analyzed with ImageJ software (RRID:SCR_003070).

### Senescence-associated β-galactosidase (SA-β-gal) staining

Cell senescence was assessed measuring SA-β-gal activity at pH 6.0 [12]. Primary patient myoblasts were fixed in 0.2% glutaraldehyde for 15 min at room temperature and stained in solution containing 1 mg/ml bromo-chloro-indolyl-galactopyranoside (X-gal), 40 mM citrid acid/sodium phosphate (pH 6), 5 mM potassium ferrocyanide, 5 mM potassium ferricyanide, 150 mM sodium chloride, 2 mM magnesium chloride. SA-β-gal^+^ cells were detected by phase contrast microscopy using Olympus Inverted Microscopes Models IX71.

### Western blot analysis

Frozen muscle samples and primary patient myoblasts were homogenized in RIPA buffer (20 mM Tris HCl pH 7.4, 150 mM NaCl, 1 mM EDTA, 1% IGEPAL, 0.1% SDS) containing protease inhibitor cocktail (Roche). Western blot analysis of equal-protein loading were performed with the following primary antibodies: rabbit polyclonal anti-TRIM32 (NBP1–33737) (1:500; Novus Biologicals); mouse monoclonal anti-Myogenin (FD5) (1:100; Abcam); mouse monoclonal anti-Myosin Heavy Chain (A4.1025) (1:500; Millipore); rabbit polyclonal anti-LC3B (1:1000; Cell Signaling), rabbit monoclonal anti-LAMP1 (D2D11) (1:1000; Cell Signaling) and rabbit polyclonal anti-GAPDH (1:2000; Sigma-Aldrich). Immunoreactivity was detected with secondary antibodies conjugated to horseradish peroxidase (1:5000; Jackson Immuno Research) and developed with SuperSignal West Femto (Thermo Fisher Scientific) using an ImageQuant LAS 4000 MiniGold System (GE Healthcare Life Sciences).

### Proliferation and differentiation assays

CD56-positive cells were seeded at 15,000 cells/cm^2^ using proliferation medium. We examined proliferation over a period of time from 0 to 8 days. The average number of cells for each time point (0, 4, and 8 days) was then calculated and the values were used to plot growth curves. When the myoblasts started to fuse, the medium was substituted with one containing 2% of FBS without growth factor to induce differentiation. We measured the fusion index 4 days after medium change by calculating the mean percentage of nuclei in myotubes, relative to the total number of nuclei (myoblasts + myotubes).

### Autophagy blockade experiment

Bafilomycin A1 (Baf-A1), an autophagosome-lysosome fusion inhibitor, inhibits autophagy by preventing the fusion of the lysosome to the autophagosome [46]. Myoblasts after 4 days in proliferation medium were incubated with 1 μM Baf-A1 (Cayman) for 6 h, followed by lysis and western blot analysis.

### Statistical analysis

Graphpad Prism software (RRID: SCR_002798) was used to analyze the data using one-way ANOVA. When the ANOVA analysis revealed significant differences, the post hoc Bonferroni’s test was used for pairwise comparisons. When parametric assumptions were not met, Kruskal-Wallis followed by the post hoc Dunn’s test were used. Data were plotted as mean ± SEM.

## Results

### Mutations in NHL, coiled-coil and RING domains of TRIM32 are found in patients with a muscular dystrophy

**Fig. 1:**
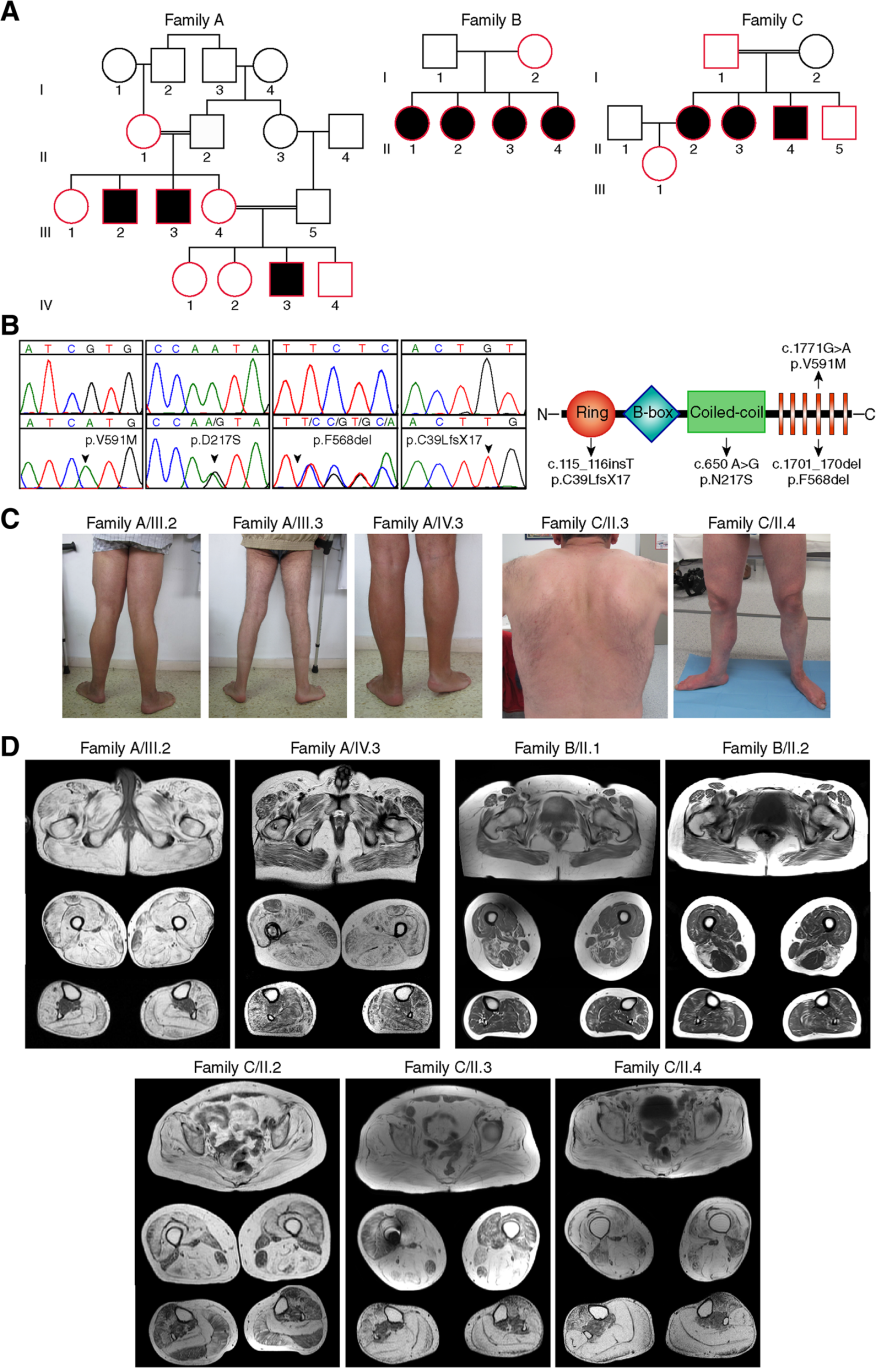
Pedigrees, genetic findings and clinical features of three families with mutations in *TRIM32*. **a** Pedigree of Spanish family (family A) having the missense c.1771G > A (p.V591 M) mutation that segregates with the disease: homozygous only in affected patients. Pedigree of Australian family (family B) presenting the missense c.650A > G (p.N217S) mutation in combination with the frameshift c.1701_1703del (p.F568del) mutation that segregate with the disease: compound-heterozygosity only in affected patients. Pedigree of Spanish family (family C) possessing the homozygous frameshift mutation c.115_116insT (p.C39LfsX17) that segregates with the disease: homozygous only in affected patients. In the non-affected siblings (red symbols) of the three pedigrees we found only one or no mutated alleles. Circles denote female members and squares male members. Double line denotes a consanguineous marriage. Solid black symbols denote affected patients. **b**. Chromatograms display the homozygous missense c.650A > G (p.N217S), heterozygous missense c.650A > G (p.N217S), heterozygous frameshift c.1701_1703del (p.F568del) and homozygous frameshift mutations in the *TRIM32* gene. Arrows indicate the location of the base change. The TRIM32 functional domains RING finger domain, B-box type 1 domain, coiled-coil region, and six NHL repeats are represented in the scheme, where arrows indicate the novel mutations location in the TRIM32 protein structure. **c**. Distal muscle involvement led to distal atrophy and ankle contractions. Patient II.3 from Family C showed mild paravertebral muscle atrophy with no scapular winging. **d**. Muscle MRI T1-weighted axial images at gluteus, thigh and calf levels showed different patterns of muscle involvement in the three families

We studied three independent families of Spanish and Australian origin with a muscular dystrophy (Fig. 1a), and identified four novel *TRIM32* mutations located in three different domains; NHL, coiled-coil and RING domains. Patients II.2, II.3 and III.3 from family A were homozygous for *TRIM32* c.1771G > A (p.V591 M) involving the fourth NHL repeat. None of the six healthy relatives studied from three different generations were homozygous for the mutation, whose frequency in gnomAD was 0.002%. Patients II.1, II.2, II.3 and II.4 from family B were compound heterozygous for c.650 A > G (p.N217S) and c.1701_1703del (p.F568del) mutations, involving the coiled-coil and fourth NHL repeat respectively. The unaffected mother was heterozygous for the c.650 A > G (p.N217S) mutation and the father was not available, thus the mutations were estimated likely to be in trans. Both mutations were very rare in the population (0.002 and 0.0003% in gnomAD respectively). Patients II.2, II.3 and II.4 from Family C were homozygous for a *TRIM32* c.115_116insT (p.C39LfsX17) mutation, involving the RING domain (Fig. 1b). Segregation analysis demonstrated that the variant was found in heterozygous state in the rest of the healthy relatives studied from different generations, and was absent from the gnomAD control population.

### Distinct clinical and radiological features are identified in all families

All patients from Family A presented with foot drop as the first manifestation in their teens. This was the only symptom for years. Over further time, they developed remarkable ankle contractions. Later, they also developed proximal and distal weakness in lower and upper limbs. The youngest patient (IV.3) showed a more severe progression, with need of support for gait in his thirties (Fig. 1c).

In Family B, there was a remarkable clinical variability. Patient II.3 presented in her twenties with foot drop and proximal upper limb weakness. At age 55, she also had proximal lower limb weakness, and at age 58 she was unable to walk unsupported. Patient II.2, came to our attention at the age of 57, after the diagnosis of her sister and although she did not have any complains, examination showed mild bilateral proximal upper and lower limb weakness. Patient II.4 showed proximal and distal lower limb weakness at age 57 and patient II.1 showed normal physical exam at age of 67.

Patients from Family C, presented with limb-girdle muscle weakness with onset between third and fourth decade and later developed distal limb weakness (Fig. 1c). Interestingly, patients II.3 and II.4 presented additional systemic manifestations typically described in BBS including hypogonadism, hearing loss, and behavioral abnormalities (depression and obsessive-compulsive disorder).

No cardiac or respiratory involvement was found in any of the examined patients.

Muscle MRI revealed different patterns of muscle involvement in the three families (Fig. 1d). Family A showed diffused fatty degeneration in the thighs and lower legs. Family B displayed a more focal pattern of fatty replacement mainly involving the biceps femoris and semimembranosus muscle in the thigh and mildly in the soleus muscle in the lower legs. Interestingly, the asymptomatic patient II.1 did show moderate fat replacement of muscles. Family C showed predominant degeneration of the gluteus muscles, the posterior compartment in the thighs, and the soleus and gastrocnemius muscles in the lower legs.

### *TRIM32* gene mutations lead to a reduced TRIM32 protein level

**Fig. 2:**
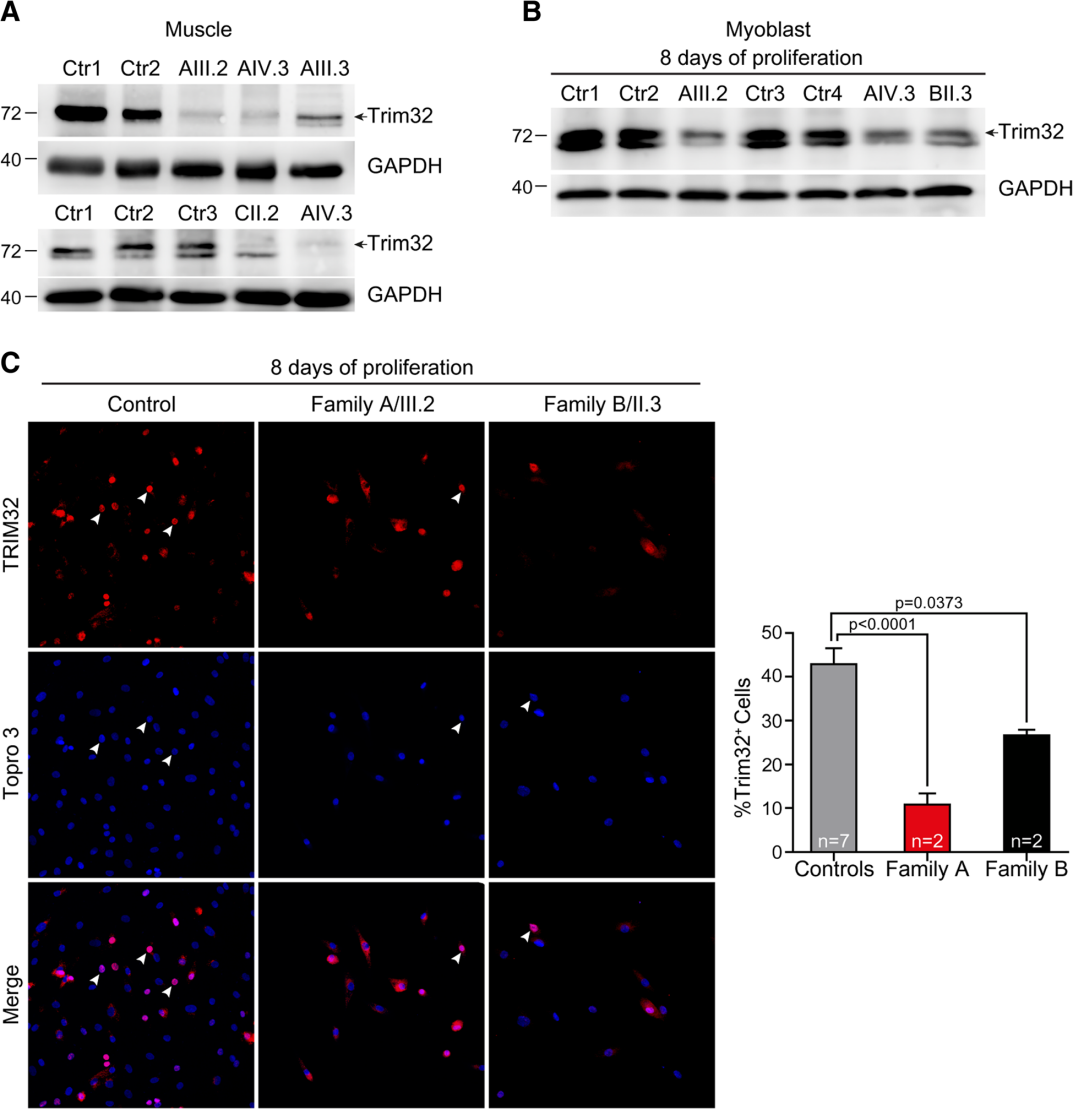
Reduced TRIM32 protein level associated with p.V591 M, p.N217S/F568del and p.C39LfsX17 mutations. **a**, **b** Western blot analysis of **a** muscle derived from biopsies of family A patients, family C patient and healthy controls, and **b** primary myoblasts from family A patients, family B patient, and healthy controls. Anti-TRIM32 antibody revealed a remarkable reduction of TRIM32 protein level in TRIM32^V591M^, TRIM32^N217S/F568del^ and TRIM32^C39LfsX17^ samples compared with controls. An anti-GAPDH blot is shown as a loading control. Arrows indicate the correct band at 72 kDa. **c**. Immunofluorescence staining and quantification of the percentage of TRIM32^+^ cells (arrowheads) in primary myoblasts at 8 days growing in proliferation medium from family A patients (*n* = 2), family B patients (*n* = 2) and healthy controls (*n* = 7). Images show TRIM32 fluorescence (red). Cells were counterstained with Topro 3 (blue) to visualize the nuclei. The percentage of TRIM32^+^ cells was significantly lower in TRIM32^V591M^ and TRIM32^N217S/F568del^ myoblasts than in controls. Data from 9 to 31 independent fields were analyzed per condition. Mean ± SEM; Kruskal-Wallis with Dunn’s multiple comparison test. Scale bar, 50 μm

At present, most *TRIM32* reported mutations are clustered in the highly conserved C-terminal NHL domain of TRIM32 and may cause conformational changes in the protein that lead to a substantial decrease in its stability. This hypothesis is supported by the reduced level of TRIM32 found in human fibroblasts isolated from LGMD2H patients carrying the homozygous p.D487N substitution and in the muscle from the mouse model T32KI (harboring the p.D489N substitution) [1, 25]. Based on these observations, we analyzed the effect of the novel mutations on the presence of TRIM32 in muscle samples from patients. Western blot revealed almost undetectable TRIM32 protein level in TRIM32^C39LfsX17^ muscle, which was an expected result because it is a frame-shift mutation resulting in a premature stop codon, which in turn should result in a severely truncated TRIM32 protein. However, we also found a remarkable reduction of TRIM32 level in TRIM32^V591M^ and TRIM32^N217S/F568del^ muscles or primary myoblasts, compared to controls (Fig. 2a-b). Nicklas et al. found that proliferating mouse myoblasts in culture displayed nuclear TRIM32 signal and a shift of TRIM32 to the cytoplasm during differentiation [35]. In human samples, immunostaining assay showed a reduced amount of proliferating myoblasts with positive signal of TRIM32 in the nuclei of TRIM32^V591M^ and TRIM32^N217S/F568del^ myoblasts (Fig. 2c). These data support that not only the frameshift in the RING domain but also the single amino acid deletion and missense *TRIM32* mutations identified in the NHL and coiled-coil domains resulted in reduced levels of TRIM32 protein.

### Mutations in TRIM32 impair proliferation and myogenic differentiation in primary myoblast

**Fig. 3:**
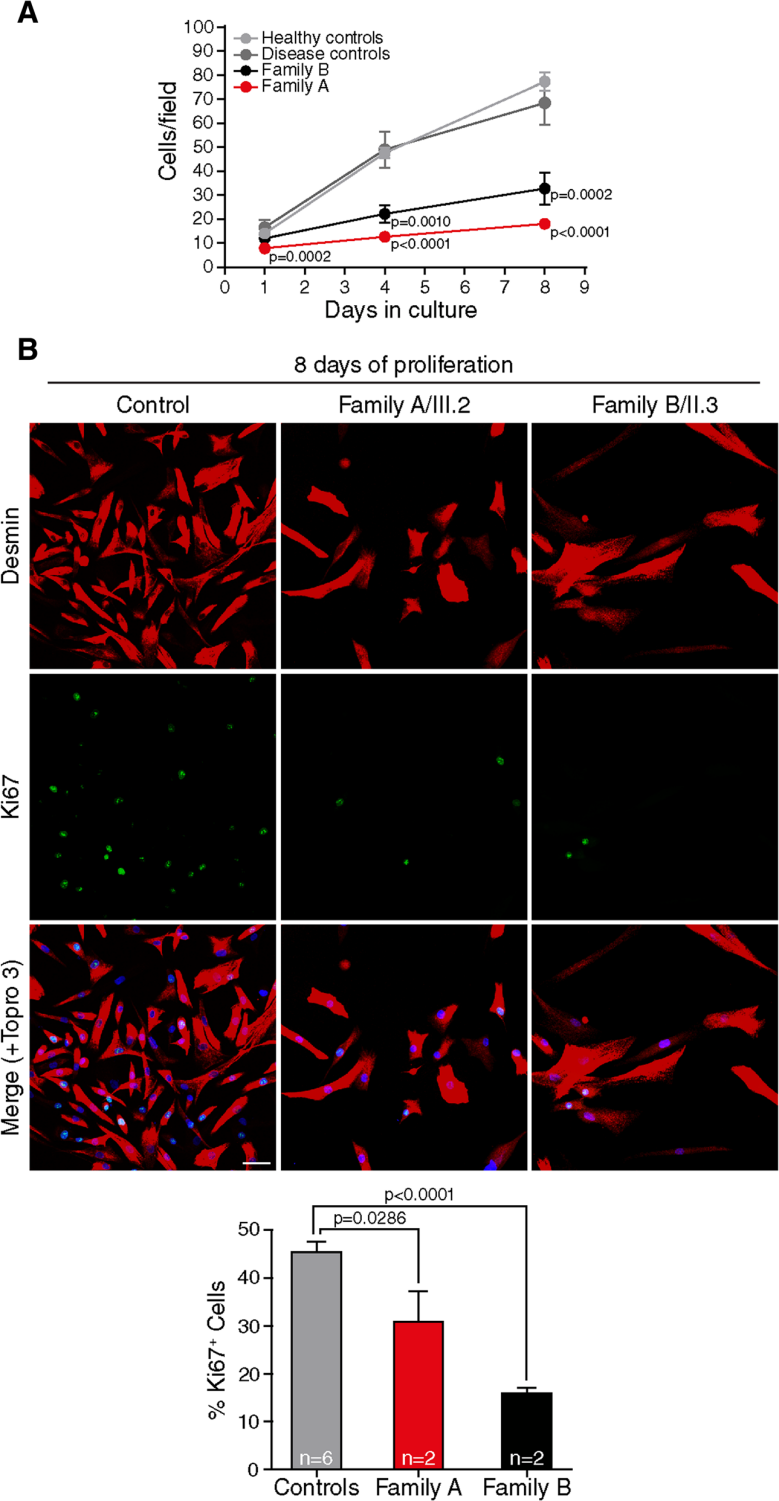
TRIM32 mutant myoblasts exhibit a decrease in cell proliferation. **a** Quantification of number of primary myoblast (proliferation rate) at 1, 4 and 8 days growing in proliferation medium from family A patients (*n* = 2), family B patients (*n* = 2), healthy controls (*n* = 6) and disease controls (2 LGMD2B, X-EDMD) (*n* = 3). The proliferation rate is reduced in TRIM32^V591M^ and TRIM32^N217S/F568del^ myoblasts compared with controls. Data from 12 to 45 independent fields were analyzed per time point. Mean ± SEM; Kruskal-Wallis with Dunn’s multiple comparisons test. **b**. Analysis of primary myoblast proliferation at 8 days growing in proliferation medium using Ki67 as a marker of dividing cells from family A patients (*n* = 2), family B patients (*n* = 2) and healthy controls (*n* = 6). Immunofluorescence showing double staining, desmin (red) and Ki67 (green). Nuclei were counterstained with Topro 3 (blue). Quantification of Ki67^+^ cells revealed a progressive decrease in the percentage of proliferating TRIM32^V591M^ and TRIM32^N217S/F568del^ myoblasts compared with controls. Data from 14 to 41 independent fields were analyzed per condition. Mean ± SEM; Kruskal-Wallis with Dunn’s multiple comparisons test. Scale bar, 50 μm

**Fig. 4:**
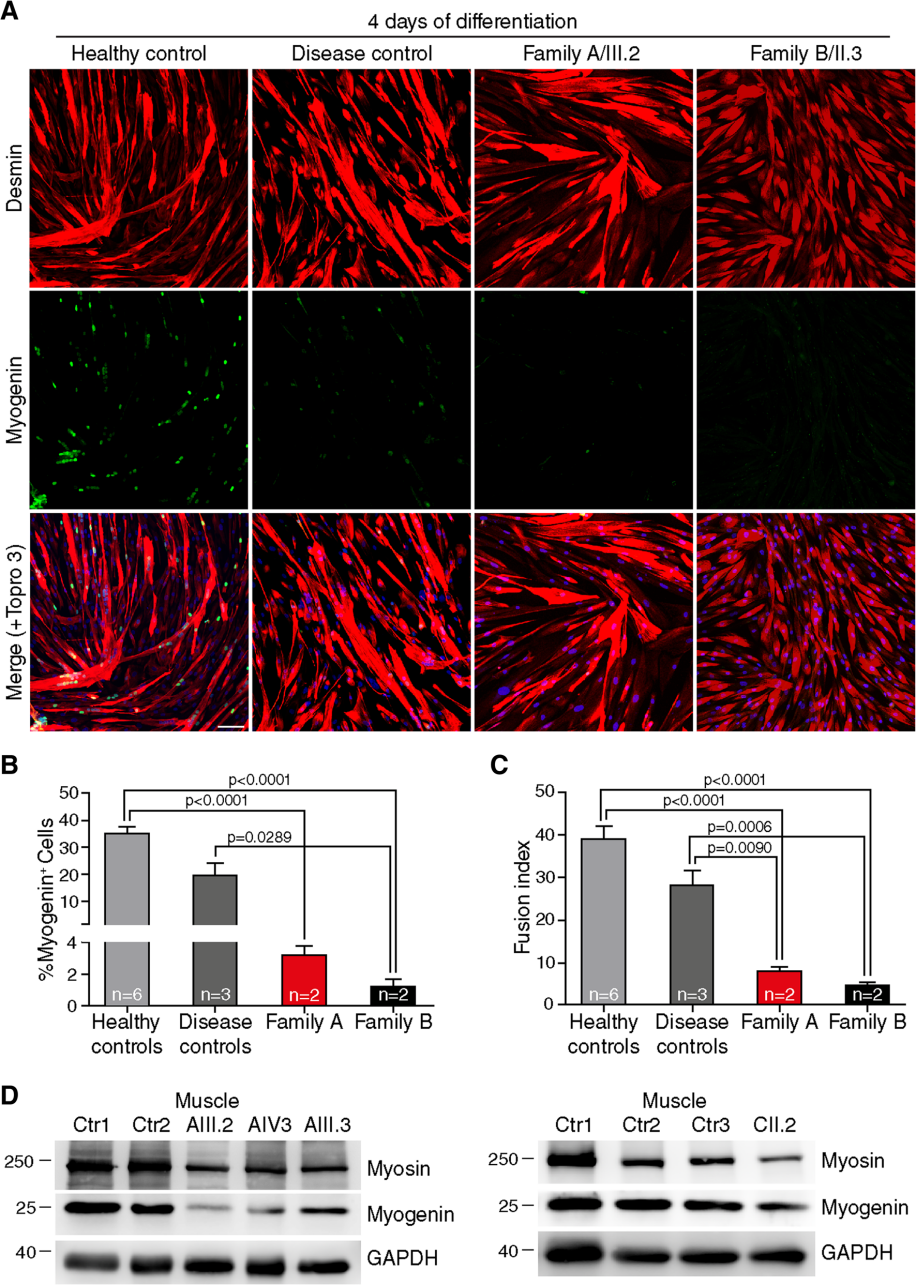
TRIM32 mutant myoblasts show impaired differentiation. **a**-**c** When primary myoblasts from family A patients (*n* = 2), family B patients (*n* = 2), healthy controls (*n* = 6) and disease controls (2 LGMD2B, X-EDMD) (*n* = 3) reached confluence, proliferation medium was replaced with differentiation medium and the myoblasts started to fuse into myotubes, which were analyzed after 4 days of differentiation. **a** Immunofluorescence showing double staining of primary myoblasts, desmin (red) and myogenin (green). Nuclei were counterstained with Topro 3 (blue). **b** The expression of myogenin and **c** the fusion index were reduced in TRIM32^V591M^ and TRIM32^N217S/F568del^ myoblasts compared with controls. Data from 10 to 32 independent fields were analyzed per condition. Mean ± SEM; Kruskal-Wallis with Dunn’s multiple comparisons test. Scale bar, 50 μm. **d** Western blot analysis of biceps muscle lysates derived from family A patients, family C patient and healthy controls. Myosin heavy chain and myogenin antibodies show reduced expression in TRIM32^V591M^ and TRIM32^C39LfsX17^ muscles compared with controls. An anti-GAPDH blot is included as a loading control

Data from T32KO mice shows that TRIM32 is necessary for proliferation and differentiation of satellite cells through the regulation of the transcription factor c-Myc [35], and that the loss of TRIM32 function deregulates these processes due to the accumulation of TRIM32 substrates involved in myoblast proliferation and myogenesis [31]. We studied whether loss of protein due to *TRIM32* human mutations alter myoblast proliferation in vitro. To address this point, primary myoblasts from patients carrying p.V591 M and p.N217S/p.F568del mutations were cultured. The cell proliferation rate (Fig. 3a) and percentage of Ki67^+^ cells (Fig. 3b) were significantly lower in mutant myoblasts compared with controls. Next, we investigated if muscle differentiation was also altered when TRIM32 is reduced. The confluent myoblasts were cultured in differentiation medium, in which myoblasts withdraw from the cell cycle, cease to divide, and start to elongate and fuse to form multinucleated myotubes. Myogenin expression (Fig. 4a-b) and fusion index (Fig. 4c) were reduced in mutant myoblasts compared with controls. These results were further supported by western blot in which we found a reduction of the differentiation markers myosin heavy chain and myogenin in TRIM32^V591M^ muscle compared with controls (Fig. 4d). TRIM32^C39LfsX17^ myoblasts showed an extremely slow growth due to the reduced proliferation that precluded the production of enough cells for these experiments. Taken together, our data suggests that the loss of TRIM32 protein due to different mutations reduced myoblast proliferation and delayed myogenic differentiation.

### TRIM32 mutations in humans lead to premature senescence of myoblasts

**Fig. 5:**
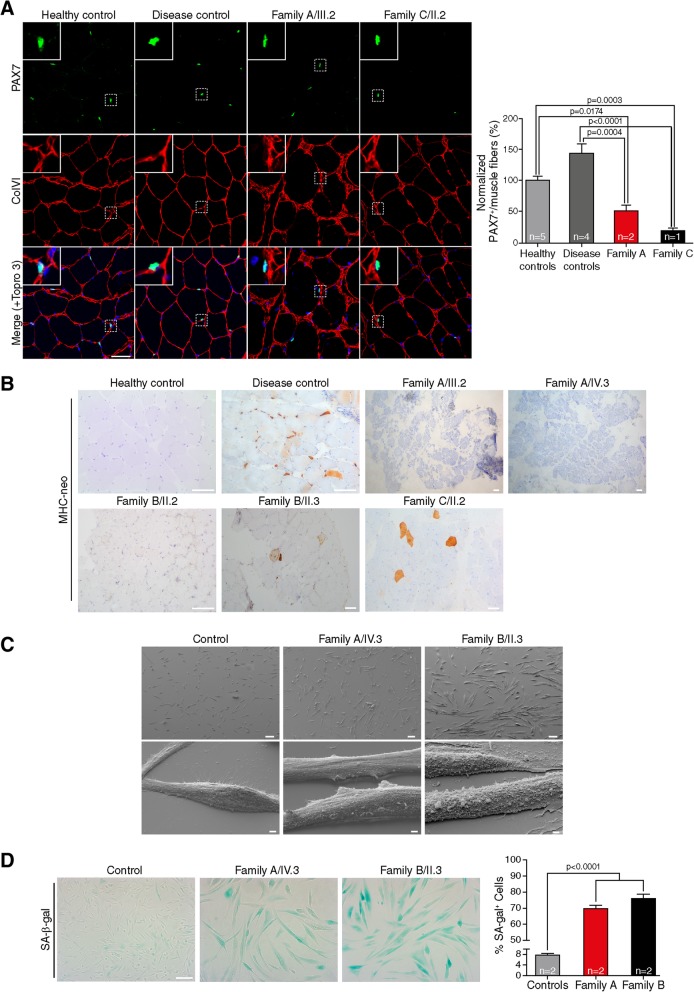
TRIM32 mutations seem to lead to premature senescence of myogenic cells. **a** Immunohistochemistry staining and quantification of Pax7^+^ satellite cells in skeletal muscle from family A patients (*n* = 2), family C patient (*n* = 1), healthy controls (*n* = 5) and disease controls (LGMD2B, LGMD1C, LGMD2N, NEM6) (*n* = 4). Immunostaining for collagen type IV (red) to show the muscle fibers, Pax7 (green) to show the satellite cells and with Topro 3 staining for the nuclei (blue). Quantification of Pax7^+^ cells revealed a significant reduction in the number of satellite cells in TRIM32^V591M^ and TRIM32^C39LfsX17^ muscles compared with controls. Data from 8 to 31 independent fields were analyzed per condition. Mean ± SEM; Kruskal-Wallis with Dunn’s multiple comparisons test. Scale bar, 50 μm. **b** Immunohistochemical staining of MHC-neo of skeletal muscle from family A patients, family B patients, healthy control and disease control (LGMD1B) revealed a large number of positive regenerating fibers in the disease control. In contrast, TRIM32 patients showed no positive cells (patients A/II.2, A/IV.3 and B/II.2) or, at most, few scattered positive cells (patients B/II.3 and C/II.2). Scale bar, 100 μm. **c** SEM images of myoblasts at 5 days growing in proliferation medium from AIV.3 and BII.3 patients, and healthy controls. TRIM32^V591M^ and TRIM32^N217S/F568del^ myoblasts were larger than control myoblasts. Higher magnification showed a reduction in the size of projections and number of filopodia of TRIM32^V591M^ and TRIM32^N217S/F568del^ myoblasts comparing to control myoblasts. Scale bars, 100 μm: lower magnification view; 2 μm: hyper magnification view. **d** Immunofluorescence staining and quantification of the percentage of SA-β-gal^+^ cells in human myoblasts after 10 days growing in proliferation medium from family A patients (*n* = 2), family B patients (*n* = 2) and healthy controls (*n* = 2). A higher increment of SA-β-gal^+^ cells was observed in TRIM32^V591M^ and TRIM32^N217S/F568del^ myoblast cultures compared to controls, supporting a premature senescence in the muscles with TRIM32 altered function. Data from 8 independent fields were analyzed per condition. Mean ± SEM; One-way ANOVA with Tukey’s multiple comparisons test. Scale bar, 100 μm

As mentioned, altered myogenesis and premature senescence are postulated to underlie myopathy in LGMD2H. Aging of muscle is characterized by functional impairment, loss of quiescence and reduction of the pool of satellite cells leading to altered regenerative capacity of the muscle [6, 45]. Additional features of senescent cells include overexpression of SA-β-gal [12], and altered morphology [36]. To study whether the reduced cellular growth and myotube formation observed in TRIM32 mutant myoblasts were associated with premature senescence, we analyzed the satellite cells pool, the degree of muscle regeneration and the existence of morphological and metabolic changes in muscle from patients with novel TRIM32 mutations. PAX7 staining (an established marker for satellite cells in adult skeletal muscle), showed severe reduction of the satellite cell pool in TRIM32^V591M^ and TRIM32^C39LfsX17^ skeletal muscles compared to controls (Fig. 5a). To evaluate the regenerative activity, muscle sections were stained with an antibody to MHC-neo, which is expressed in regenerating myofibers [40]. Unlike the disease control muscle, no staining of MHC-neo or few scattered positive myofibers was detected in muscle samples with mutant TRIM32 (Fig. 5b).

Morphological characterization of TRIM32-mutant myoblasts was examined by SEM, showing that mutant myoblasts were larger in size and flatter than controls. In addition, TRIM32-mutant myoblasts had smaller projections and a reduction in filopodia, which could restrict the cellular mobility compared to control myoblasts (Fig. 5c). To determine possible metabolic changes of TRIM32-mutant myoblasts, we quantified the number of cells positively stained for SA-β-gal activity. A higher percentage of SA-β-gal positive myoblasts was observed in TRIM32^V591M^ and TRIM32^N217S/F568del^ myoblasts compared with control myoblasts (Fig. 5d). These results suggest that TRIM32-mutant myoblasts may undergo premature senescence.

### Rimmed vacuoles and signs of autophagy activation are usual findings in muscle biopsies from patients with mutations in *TRIM32*

**Fig. 6:**
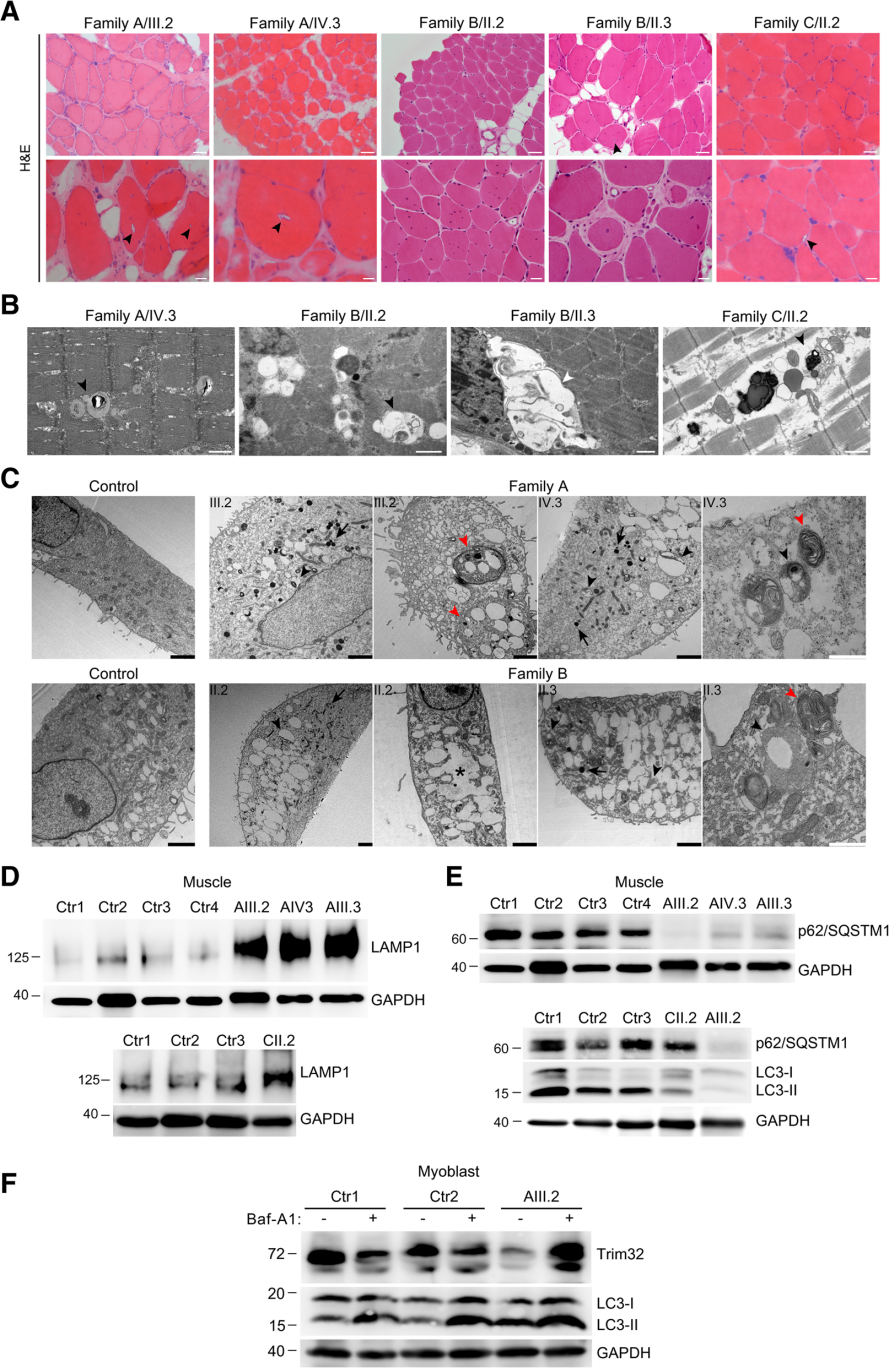
TRIM32 mutations increase autophagy activity. **a** H&E staining of skeletal muscle from family A patients, family B patients and family C patient showed internalized nuclei, fibers splitting, fibrosis and atrophy. In some fibers small rimmed vacuoles were found (black arrowheads). Scale bars, 50 μm: lower magnification view; 20 μm: higher magnification view. **b** TEM analysis of skeletal muscle from family A patient, family B patients and family C patient revealed membrane-surrounded vacuoles (white arrowheads), some of them containing slightly electron-dense amorphous material (black arrowheads).**c**. TEM images of myoblasts at 8 days growing in proliferation medium from family A patients, family B patients and healthy controls. Lysosome load and autophagic vacuoles were increased in TRIM32^V591M^ and TRIM32^N217S/F568del^ myoblasts compared to control myoblasts. Lysosomes (black arrows), autolysosomes filled with partially degraded materials (black arrowheads), multi-vesicular/lamellated structures (red arrowheads), and fusion of electron-lucent vacuoles generating large vacuoles in the cytoplasm space (asterisk). Black scale bars, 2 μm; white scale bars, 1 μm. **d** Western blot analysis of muscle biopsy derived from family A patients, family C patients and healthy controls. Anti-LAMP1 (lysosome marker) revealed an increment of LAMP1 protein level in TRIM32^V591M^ and TRIM32^C39LfsX17^ muscles compared with controls. An anti-GAPDH blot was used as a loading control. **e** Western blot of muscle biopsy from Family A and C patients. Anti-p62/ SQSTM1 and LC3-II showed reduced level in TRIM32-related myopathy patients compared to healthy controls. Although P62/SQSTM1 level in the only muscle sample available from Family C was not so reduced as in Family A, indeed it was slightly reduced since the GAPDH indicates more protein being present in the lane than the controls. An anti-GAPDH blot was used as a loading control. **f** Western blot experiments of primary myoblasts from AIII.2 patient and healthy controls incubated with vehicle or with 1 μM Baf-A1 for 6 h. Anti-TRIM32 antibody revealed that TRIM32^V591M^ protein level was rescued after treatment with Baf-A1. Anti-LC3 (autophagy marker) showed that degradation of LC3-II was partially inhibited, whereas LC3-I, as expected, was not affected in the presence of Baf-A1. Anti-GAPDH levels were used as loading control

Histological analysis of the muscle biopsies from patients showed a severe dystrophic pattern encompassing internalized nuclei, endomysial fibrosis, and necrotic and atrophic myofibers. Small vacuoles containing basophilic material were observed throughout the sarcoplasm in scattered myofibers from all muscle biopsies examined, but no empty vacuoles were identified by optical microscopy (Fig. 6a), unlike previously described cases of *TRIM32* mutations [19, 28, 42]. Ultrastructural analysis by TEM showed the vacuoles are membrane-bound, with many containing amorphous granular material (Fig. 6b).

The presence of membrane-bound vacuoles containing cytoplasmic degradation products suggests altered autophagy. We examined the cytoplasmic content of TRIM32-mutant myoblasts by TEM, and increased numbers of lysosomes and autophagic vacuoles were detected in TRIM32^V591M^ and TRIM32^N217S/F568del^ myoblasts compared with controls (Fig. 6c). We observed numerous electron-dense vacuoles consistent with autolysosomes and many multi-vesicular/lamellated structures. In addition, immunoblotting for LAMP1, a lysosomal marker, revealed increased lysosomal content in TRIM32^V591M^ and TRIM32^C39LfsX17^ muscle compared with controls (Fig. 6d). P62/SQSTM1 is a marker of autophagic activity because it directly binds to LC3-II in the autophagosome membrane. Both LC3-II and P62/SQSTM1 are selectively degraded by autophagy. Western blot demonstrated a striking reduction of p62/SQSTM1 level in muscle from the three patients from family A (Fig. 6e, upper panel), while in the only muscle sample available from family C was slightly reduced compared to controls (Fig. 6e, lower panel). Levels of LC3-II were also reduced in muscles from family A and C (Fig. 6e, lower panel). On the other hand, patient myoblasts in culture showed an increase of LC3-II in the basal state compared to controls, which further increases when fusion between autophagosomes and lysosomes was inhibited in the presence of Baf-A1 (Fig. 6f). Together, these results suggest that increased autophagic flux is present in TRIM32-related myopathy.

### Mutated TRIM32 is degraded by autophagy

Previous studies have shown that the EI24 autophagy-associated transmembrane protein is involved in autophagy-mediated degradation of RING E3 ubiquitin ligases [11]. As TRIM32 is an E3 ubiquitin ligase, we analyzed whether the degradation of TRIM32 mutated protein was dependent on autophagy. For this experiment we used TRIM32^V591M^ myoblasts. After autophagy was inhibited using Baf-A1, western blot analysis of primary myoblasts lysates showed that mutant TRIM32 protein level was efficiently rescued (Fig. 6f). This result supports that TRIM32^V591M^ is degraded via the autophagy pathway.

## Discussion

In an effort to establish if the muscle from patients with muscular dystrophy due to *TRIM32* mutations shows evidence for the pathogenic mechanism postulated from mouse models of this disease, we studied the skeletal muscle and myoblasts from three families with a muscular dystrophy due to mutations involving the NHL, coiled-coil and RING domains of TRIM32. A common consequence of all the mutations was the remarkable reduction of TRIM32 protein muscle level, regardless of the type of mutation (frameshift, single amino-acid deletion or missense) and the location within the protein. This supports a similar underlying pathomechanism for all of the mutations. We also demonstrated reduced myoblast proliferation/differentiation and premature senescence, reproducing the findings that had been seen in animal models [23, 25, 31, 35]. We have also shown evidence of autophagy activation, probably involved in the degradation of the mutated TRIM32.

Experiments in the T32KO mice pointed at TRIM32 as a protein involved in the control of myogenic cell proliferation/differentiation and satellite cell senescence due to the accumulation of the TRIM32 substrates NDRG2 and PIAS4 in skeletal muscle, secondary to the failure of their ubiquitination by TRIM32 [23, 31]. After exploring these processes in myoblasts with mutations affecting different TRIM32 domains that causes loss of protein, our results identified a significant delay in proliferation and differentiation. The features of premature senescence demonstrated in muscles from patients with *TRIM32* mutations, including a reduced pool of satellite cells and increased expression of SA-β-gal, are plausible consequences of the altered myogenic process. An alteration of myogenesis has been described in other hereditary muscle disorders, as a primary mechanism [43] or as a concomitant effect associated with, or in the absence of premature senescence [2, 9, 38]. In the case of TRIM32 the accumulation of several substrates involved in different biological processes and the frequent finding of rimmed vacuoles, support that, in addition to the altered myogenesis, other pathways could be playing a pathogenic role.

The presence of increased amounts of autophagic vacuoles, lysosomes and a high level of LAMP1 in muscle with mutated TRIM32 pointed to an alteration in autophagy. Degradation of p62/SQSTM1 is a widely used marker to monitor autophagic activity because it directly binds to LC3-II in the autophagosome membrane and is degraded by autophagy. Decreased level of p62/SQSTM1 in muscle form TRIM32-myopathy patients supports an increased autophagy. LC3-II is also degraded by the lysosome along with the autophagosome content, so a decrease in LC3-II over time provides an estimate of the autophagic flux. LC3-II in muscle from our patients showed decreased LC3-II, which also supports increased autophagy, but LC3-II amount at a given time point does not necessarily estimate the autophagic activity. However, the increase of LC3-II in mutant myoblast culture after Baf-A1 treatment supports increased autophagic flux. The autophagy in the muscle with TRIM32 mutations could be activated by different triggers. First, it is well established that autophagy is essential to maintain the population of satellite cells and their function [16, 17], and that failure of autophagy in aged satellite cells causes senescence. It has been demonstrated that the experimental induction of senescence leads to mTOR downregulation and autophagy activation [32, 47]. Therefore, the premature senescence in LGMD2H muscles could lead to increased autophagy perhaps offsetting some of the effects of the TRIM32 alteration. On the other hand, the accumulation of substrates that are no longer ubiquitinated due to loss of the mutated TRIM32 protein and therefore not degraded by the proteasome machinery, could activate autophagy as an alternative degradation pathway [41]. Further investigations could clarify this point.

In mature healthy muscle, TRIM32 level is normally low because it seems to be restricted to satellite cells [23, 35]. This makes detecting the protein in skeletal muscle challenging. Nevertheless, we found that the TRIM32 protein level was markedly reduced in skeletal muscle from patients with the missense mutations, single amino acid deletion, or frameshift mutation compared to controls. Data about TRIM32 protein level in muscle of patients with *TRIM32* mutations is scarce. Expected loss of protein by immunoblot was reported in a patient compound heterozygous for a large deletion that removes the whole *TRIM32* gene and a frameshift mutation, while heterozygous relatives for either of the mutations showed normal TRIM32 level [5], which is the same as we found in TRIM32^C39LfsX17^ muscle. To date, no reported data exist about the level of TRIM32 in muscle from patients with *TRIM32* missense mutations. The T32KI mouse model carrying the Hutterite missense mutation p.D489N shows a severe reduction of TRIM32 by immunoblot, and the same neuromuscular phenotype as *Trim32*-null mice [25]. Our results of profound reduction of TRIM32 level associated with novel missense mutations are fully consistent with previous studies demonstrating a markedly reduced amount of TRIM32 in primary fibroblasts cultured from LGMD2H patients homozygous for the p.D487N substitution [1]. This finding suggests that the mutant TRIM32 proteins might undergo degradation. Thus, reduced level of the protein detected by immunoblot could support the pathogenic role of future novel *TRIM32* missense mutations classified as variants of unknown significance (VUS), in the appropriate clinical context [21].

Mutations in the NHL, B-box and coiled-coil domains of TRIM32 have been previously described [7, 21], but to date mutations in the RING domain had not been reported. Here, we report that all the mutations are associated with a loss of protein leading to a muscular dystrophy. The presenting features were highly variable regarding severity, proximal/distal distribution and muscle MRI pattern, but with common histological features in all cases. Different phenotypes produced by mutations in *TRIM32*, even in the same region of the gene, has similarly been reported in other myopathies associated with mutation in other genes such as *MYH7* [27]. Strikingly, two of the three patients with the mutation p.C39LfsX17 in the RING domain presented, in addition to the muscle dystrophy, hypogonadism, hearing loss and behavioral abnormalities, symptoms typically described in the BBS previously associated with a mutation in the B-box domain [3, 10]. BBS is a rare ciliopathy characterized by multisystemic manifestations (retinal degeneration, obesity, polydactyly, renal abnormalities, hypogonadism, behavioral abnormalities, among others), but no muscle weakness. Only one family with BBS and TRIM32 mutation in the B-box domain has been described, and symptoms of BBS have not been previously described in patients with TRIM32 muscular dystrophy. The variable phenotypes in patients with mutations in TRIM32 are most likely explained by modifying factors that have yet to be identified and would have to be studied in as large a cohort of TRIM32 patients as could be assembled.

## Conclusion

Our results demonstrate a common pathogenic pathway in the development of muscular dystrophy associated to TRIM32 mutations which lead to loss or reduction of the protein. The main effect of TRIM32 mutations is consistent with alterations of myogenesis which induce a diminished pool of satellite cells and accelerate the senescence of skeletal muscle. We also demonstrated signs of autophagy activation, that could be the response to different triggers but probably collaborating in the pathogenic process. We also provide clinical evidence of TRIM32-related myopathy patients presenting muscular weakness together with BBS clinical manifestations, but to elucidate responsible factors additional studies must be carried out. The identification of an increasing number of VUS, especially missense variants, complicates the diagnostic process of genetic disorders. In the case of *TRIM32* mutations, besides myoblast culture to demonstrate reduced proliferation and differentiation, which is not always available, we propose immunoblot to characterized the effect of novel candidate pathogenic variants on TRIM32 protein level.

## Abbreviations

Baf-A1: Bafilomycin A1
BBS: Bardet-Biedl syndrome
EDMD: Emery-Dreifuss muscular dystrophy
LGMD: Limb-girdle muscular dystrophy
MRI: Magnetic resonance imaging
NDRG2: N-myc down-regulated protein 2
NEM: nemalin myopathy
PIAS4: E3 small ubiquitin-related modifier (SUMO) ligase
SEM: Scanning Electron Microscopy
STM: Sarcotubular myopathy
T32KI: *Trim32* knock-in
T32KO: *Trim32* knockout
TEM: Transmission Electron Microscopy
TRIM32: Tripartite motif-containing protein 32
VUS: Variants of unknown significance
